# Global Action on Biodiversity May Hinge on Genetic Data Sharing Agreement

**DOI:** 10.1002/ggn2.202200031

**Published:** 2022-12-22

**Authors:** Jacqueline Batley, Andrew L. Hufton, Guilherme Oliveira, Rajeev K. Varshney

**Affiliations:** ^1^ School of Biological Sciences The University of Western Australia Crawley WA 6009 Australia; ^2^ Wiley‐VCH GmbH 69469 Weinheim Germany; ^3^ Instituto Tecnológico Vale Belém 66055‐090 Brazil; ^4^ Centre for Crop & Food Innovation State Agricultural Biotechnology Centre Food Futures Institute Murdoch University Murdoch WA 6150 Australia

This month, parties to the Convention on Biological Diversity (CBD) are meeting in Montreal with the aim of concluding negotiations on an important new action plan for global biodiversity conservation, known as the post‐2020 Global Biodiversity Framework (GBF). In these negotiations, genetic data from plants, animals, fungi and microorganisms, known as digital sequence information (DSI) in policy circles, has emerged as a central source of tension. A number of parties are demanding that benefits arising from the use of these genetic data be better shared with the countries where the genetic material was collected.

The Nagoya Protocol, a component of the CBD, recognized the right of countries to share in the benefits derived from their nation's genetic resources, and established a framework by which countries can regulate and track the use of physical “genetic resources” (i.e., biological samples, strains, plant lines, etc., containing genetic material). This framework, however, is complex and, in the opinion of many, has proven inefficient at driving meaningful benefit sharing.^[^ ^1^ ^]^

Researchers and other stakeholders have raised serious concerns about applying such a framework to DSI.^[^ ^2^, ^3^ ^]^ Some of the proposals on the table could spell an end to the culture of open sequence sharing that has defined non‐human genetics research for decades, and which is widely agreed to have massive positive effects on research progress and economic value creation. A poorly developed solution could therefore have a negative impact on biodiversity research that is crucial to the aims of the CBD. Representatives of indigenous peoples and local communities have also been active in the discussions on this topic and argue that the rights and roles of their communities must be respected in any final agreement.^[^ ^4^ ^]^

That this issue alone could stymie global biodiversity conservation efforts is not in doubt. Talks in August on a major ocean biodiversity treaty failed to make progress because of lack of agreement on DSI,^[^ ^5^ ^]^ and African negotiators have warned that they will not agree to a GBF that lacks a concrete solution to DSI.^[^ ^6^ ^]^ The issue has also proven contentious in a recent meeting of the Governing Body session of the International Treaty on Plant Genetic Resources for Food and Agriculture.^[^ ^7^ ^]^ It is clear that an effective benefit sharing solution must be part of any global action plan to conserve biodiversity.

But grounds for optimism remain. Scientists and major research organizations are arguing that it is possible to build a solution that will drive benefit‐sharing, protect open science and promote biodiversity conservation.^[^ ^4^, ^8^, ^9^ ^]^ An open letter expressing these principles, organized by the DSI Scientific Network, has been signed by over a thousand researchers and more than 30 scientific organizations and institutions from around the globe.^[^ ^10^ ^]^

We, writing both as scientists and on behalf of *Advanced Genetics*, encourage researchers to raise their voices on this crucial issue and speak out for sensible and equitable solutions that will preserve scientific data sharing. We must defend the collaborative nature of modern research, because only together can we address the major challenges facing our planet.

## Conflict of Interest

A.L.H., G.O. and R.K.V. are unpaid members of the DSI Scientific Network.
